# Aspirin with or without statin in the treatment of endotheliitis, thrombosis, and ischemia in coronavirus disease

**DOI:** 10.1590/0037-8682-0472-2020

**Published:** 2020-09-21

**Authors:** Francisco Kleyton Zacarias Florêncio, Maiza de Oliveira Tenório, Aluísio Roberto Andrade Macedo, Sandro Gonçalves de Lima

**Affiliations:** 1 Hospital Metropolitano Sul Dom Hélder Câmara, Cabo de Santo Agostinho, PE, Brasil.; 2 Universidade Federal de Pernambuco, Centro de Ciências Médicas, Recife, PE, Brasil.; 3 Universidade Federal de Pernambuco, Hospital das Clínicas, Serviço de Cardiologia, Recife, PE, Brasil.; 4 Universidade Federal de Pernambuco, Departamento de Clínica Médica, Recife, PE, Brasil.

**Keywords:** COVID-19, Aspirin, Thrombosis, Endothelium, Hydroxymethylglutaryl-CoA Reductase Inhibitors

## Abstract

**INTRODUCTION:**

In the genesis of coronavirus disease (COVID-19), there is a process of endotheliitis associated with thrombotic changes, no studies have reported the use of acetylsalicylic acid (ASA) as a possible therapeutic approach. Statins could potentiate the ASA therapy.

**METHODS:**

This is a series of 14 cases with a laboratory-confirmed diagnosis of COVID-19. All patients underwent the ASA therapy. Those who had risk factors for vascular disease also underwent the high-potency statin therapy. When symptoms were totally or practically resolved, patients were discharged and advised to continue medications for a complementary time, according to the clinical evolution of each patient.

**RESULTS:**

The mean age of monitored patients was 48.6 years. A total of 78.6% patients presented with at least one comorbidity, which could have contributed as a risk factor for a poor prognosis in the evolution of COVID-19. Four patients had secondary bacterial infections; three patients needed hospitalization. None of the cases progress to stage III, and all patients had remission of symptoms, with 100% survival.

**CONCLUSIONS:**

the process of endothelial dysfunction in COVID-19 involves disseminated thrombosis, initially microvascular and later expansion into larger vessels. ASA could act as a secondary prophylaxis and prevent thrombosis from developing and reaching stage III of the disease. As this was a case series, we cannot provide definitive conclusions; however, this study allows us to formulate hypotheses and support clinical trials to evaluate benefits of the ASA therapy in the treatment of COVID-19.

## INTRODUCTION

Coronavirus disease (COVID-19) is a new disease, with no specific treatment or vaccines^1^. Hydroxychloroquine, remdesivir, and immunoglobulins are some drugs that are currently being studied in clinical trials worldwide^1^. However, none of them have been recognized for their clinical use in COVID-19.

Recent studies have suggested that in COVID-19, there is a process of multisystemic endotheliitis^2^ associated with the formation of microthrombi^3^, with a final evolution to diffuse, multiorgan ischemia^2^, which may be responsible for multiple organ dysfunctions, unfavorable clinical development, and a high mortality rate in severe cases. Ackermann et al., in autopsies of patients who had died of respiratory failure due to COVID-19, demonstrated a severe endothelial lesion associated with the presence of intracellular virus, reinforcing theories that disease progression is related to endothelial dysfunction associated with disseminated microthrombi^2^. 

In the genesis of COVID-19, there is a process of endotheliitis associated with thrombotic changes, but no studies have reported the use of acetylsalicylic acid (ASA), a well-known antiplatelet agent that acts by inhibiting cyclooxygenase 1 (COX-1)^4^, as a possible therapeutic approach. Some studies have reported that statins play an important role in protecting the endothelium^5^ ^,^ ^6^, in a manner that could potentiate the ASA therapy in COVID-19**.**

The present study is a case series of patients with mild to moderate COVID-19, who received a therapeutic approach with ASA associated or not associated with statin to treat endotheliitis and prevent thrombotic events.

## METHODS

This was a case series of 14 patients with a laboratory-confirmed diagnosis of COVID-19, treated and monitored between May and June 2020. The diagnosis was confirmed with reverse-transcriptase polymerase chain reaction, serology, or rapid tests. Patients presented with mild to moderate COVID-19.

All patients underwent the ASA therapy, with an initial dose (“loading dose”) of 200 to 300 mg, followed by daily doses of 100 mg until the 15^th^ or 21^st^ day of persistent or worsening symptoms. Patients who were already on the antiplatelet therapy (single or double therapy) continued their medications at the usual dose. In addition, patients who had risk factors for vascular disease, such as hypertension, diabetes, active or passive smoking, obesity (body mass index > 30 kg/m^2^), also received high-potency statin therapy (atorvastatin 10 to 20 mg). Patients who were already taking high-potency statins continued with their medications, while the prescriptions of patients on low-potency statins were changed to atorvastatin.

In addition to the aforementioned therapy, 13 patients received azithromycin (daily doses of 500 mg for 5 days) and nine took ivermectin (a single dose of 6 mg per 30 kg body weight). They were also instructed to undergo a home support therapy, which consisted of prone positioning for 2 h, thrice a day, in addition to using handmade expiratory positive airway pressure. 

 Patients with dyspnea or worsening asthenia from the 7^th^ to 10^th^ day of the disease received low-dose corticosteroid therapy for 4 days. Those who presented with bacterial infections were treated with broad-spectrum antimicrobials. 

When symptoms were totally or practically resolved, patients were discharged and/or advised to continue their medications for an additional period according to the clinical development.

## RESULTS

Patients included seven men and seven women, with a mean age of 48.6 years (13 to 81 years). The mean age of women was 51 years and of men was 46.1 years.

A total of 11 (78.6%) patients presented with at least one comorbidity, which could have contributed as a risk factor for a poor prognosis in the evolution of COVID-19. Six (42.9%), four (28.6%), and seven (50%) patients presented with hypertension, diabetes mellitus, and obesity or overweight. 

Nine patients with dyspnea or worsening asthenia from the 7^th^ to 10^th^ day received low-dose corticosteroid therapy (oral prednisone 0.5 mg/kg/day or class equivalent) for 4 days. Four (28.6%) patients presented with bacterial infections of the respiratory (three patients) or urinary tract (one patient) and were successfully treated with broad-spectrum antimicrobials after the first cycle. Three (21.4%) patients were hospitalized and discharged within 2 weeks. 

Details regarding the clinical status, therapies, and evolution of each patient are presented in Table 1.

**TABLE 1:** Clinical and therapeutic description of patients

AGE (in years)	COMORBIDITIES/HISTO RY	TREATMENT	OUTCOMES (from the	COMPLICATIONS
			beginning of follow-up to	
			being discharged)	
37	None	ASA^a^	Oligosymptomatic^e^ on the 10^th^ day	None
48	Asthma and hypothyroidism	ASA^a^ + atorvastatin	Oligosymptomatic^e^ on the 9^th^ day	None
41	Asthma	ASA^a^ + atorvastatin	Asymptomatic on the 10^th^ day	None
39	Hypertension, obesity	ASA^a^ + atorvastatin + oral *amoxicillin (875 mg) potassium clavulanate* (125 mg) q8h for 7 days	Oligosymptomatic^e^ on the 4^th^ day	RTI^b^
58	Hypertension, diabetes mellitus, obesity	ASA^a^ habitual^c^ + atorvastatin + oral ceftriaxone 2 g/day intravenous for 6 days + azithromycin 500 mg/day for 5 days	Oligosymptomatic^e^ on the 2^nd^ day	RTI^b^ + hospitalization
52	Diabetes mellitus, coronary artery disease, overweight	ASA^a^ habitual^c^ + clopidogrel habitual^c^; sinvastatin replaced with atorvastatin	Asymptomatic on the 10^th^ day	None
55	History of non-Hodgkin lymphoma	ASA^a^ + atorvastatin	Oligosymptomatic^e^ on the 7^th^ day	None
55	Hypertension, obesity	ASA^a^ + atorvastatin	Asymptomatic on the 7^th^ day	None
81	Hypertension, diabetes mellitus, ex-smoker	ASA^a^ + atorvastatin + IV ceftriaxone 2 g/day for 10 days + oral azithromycin 500 mg/day for 5 days	Asymptomatic on the 21^st^ day	RTI^b^+ hospitalization
36	Overweight, ex-smoker, circulation problem in the lower limbs	ASA^a^	Asymptomatic on the 14^th^ day	None
13	None	ASA^a^	Asymptomatic on the 11^th^ day	Hospitalization
63	Hypertension	ASA^a^ + atorvastatin	Oligosymptomatic^e^ on the 7^th^ day	None
60	Hypertension, diabetes mellitus, dyslipidemia, obesity, lower limb varicose veins, ex-smoker	ASA^a^+ rosuvastatin habitual^b^+ oral nitrofurantoin 100 mg q6h for 7 days	Asymptomatic on the 17^th^ day	UTI^d^
43	Overweight	ASA^a^ + atorvastatin	Asymptomatic on the 14^th^ day	None

Clinical, therapeutic, and evolutionary details of each patient Keys: **(a) ASA**: acetylsalicylic acid; (**b) RTI**: respiratory tract infection; **(c) Habitual**: medication that the patient already used routinely before the first visit and was maintained for the treatment of COVID-19; **(d) UTI**: urinary tract infection **(e) Oligosymptomatic**: patient presents with residual cough or some degree of olfactory change, but without dyspnea.

None of the cases progressed to stage III, and all patients had significant remission of symptoms, i.e., they became asymptomatic or oligosymptomatic (residual cough or some degree of olfactory change but without dyspnea), thereby meeting the discharge criteria. There was 100% survival. The longest follow-up period continued until the 30^th^ day after the onset of symptoms.

We observed no hemorrhagic complications or other adverse effects of the treatment.

## DISCUSSION

ASA is a well-established medication, used in situations such as acute myocardial infarction (AMI)^7^ ^,^ ^8^ and ischemic stroke. It is also used in the secondary prevention of AMI in patients with coronary artery disease and in preventing specific hypertensive disorders of pregnancy and/or pre-eclampsia^9^.

Histopathological studies have shown that COVID-19 involves endothelial injury and diffuse and multisystemic endotheliitis^2^ ^,^ ^10^. This process occurs because severe acute respiratory syndrome coronavirus 2 uses angiotensin-converting enzyme 2^2^ to enter the cell, which is widely present in both type II pneumocytes and the vascular endothelium^10^. This infection in the endothelial cells may result in a generalized endothelial dysfunction associated with the apoptosis process^10^. The endothelium is essential to maintaining systemic homeostasis and vascular tone owing to its significant endocrine, paracrine, and autocrine activities^10^ ^,^ ^11^. Thus, endotheliitis may lead to systemic microcirculatory dysfunction, thereby explaining the multiple manifestations and clinical sequelae in patients with this disease^10^. This process may progress to disseminated thrombosis, initially microvascular, and future expansion into larger vessels, until it greatly obstructs the circulation of several organs, leading to multisystemic ischemia. 

The work of Rudolf Virchow, developed in the 19^th^ century, provides the guiding principles of the prophylaxis, diagnosis, and therapy of deep vein thrombosis (DVT). The components of Virchow's triad (endothelial changes, blood stasis, and predisposing factors for DVT)^12^ appear to be present in COVID-19, where it is possible to identify endothelial injury, followed by diffuse and multisystemic endotheliitis and the formation of peripheral microthrombi. The inflammatory, humoral, and cellular processes cause microcirculatory stasis, which in association with diffuse endothelial lesions in patients with risk factors may evolve to small microvascular thrombotic processes, which may be disseminated and multisystemic.

Microvascular thrombosis may lead to a feedback process consisting of successive inflammatory waves, progressively more intense, with more disseminated blood stasis and more multisystemic thrombosis, in increasingly larger vessels, until the blood circulation is compromised and multisystemic ischemia sets in, causing multiple organ failure and death. Patients with vascular disease at different sites may be more severely affected, as they have a more fragile endothelium and are more prone to endothelial injury and endotheliitis^13^ ^-^ ^17^, and consequent blood stasis. Thus, it is possible to divide the disease into three well-defined stages based on the pathophysiological process: viral endotheliitis, progressive thrombosis, and diffuse ischemia (Table 2). 

**TABLE 2:** Pathophysiological stages of the evolution of coronavirus disease.

STAGES	DESCRIPTION
Viral endotheliits	● diffuse multisystemic endothelial lesion caused by the virus
	● diffuse endotheliitis
	● microvascular stasis due to endotheliitis
Progressive thrombosis	● platelet aggregation, microvascular thrombosis
	● inflammation and thrombosis of larger vessels, intravascular coagulation;
Diffuse ischemia	● multisystemic ischemia, SIRS^a^ and DIVC^b^
	● refractory shock, MOF^c^ and death

Each stage of disease progression is presented on the left, with the main events listed on the right. **(a) SIRS**: Systemic inflammatory response syndrome; **(b) DIVC**: Disseminated intravascular coagulation; **(c) MOF**: Multiple organ failure.

The state of procoagulation, which exists in patients with COVID-19, is reflected by the high level of D-dimer, prolonged prothrombin time, and thrombocytopenia^18^ ^,^ ^19^. These thrombi, which exist in COVID-19, are related to both platelet aggregation and thrombin-mediated coagulation. Zhou et al., in a study involving 191 patients, reported that D-dimer levels of 1 µg/mL and above on admission were associated with higher mortality, reflecting the action of the coagulation system in the poor prognosis of the disease^20^. Oxley et al. reported five cases of stroke due to thrombus formation in young patients with COVID-19 in New York^21^. Thus, in COVID-19, there seems to be an interaction between the endothelium and the immune system, where the degree of aggression and magnitude of the inflammatory response determine the manner in which the disease progresses^3^. Therefore, the use of drugs focused on modulating this interaction may also be a treatment method for COVID-19.

Studies have already indicated the possible benefit of using heparin in moderate to severe COVID-19^18^. Liu et al. reported using dipyridamole as a possible beneficial therapeutic approach in patients with COVID-19, suggesting that prophylactic anticoagulation should be considered to prevent the disease from progressing to a more critical condition^22^. Within this context, considering that ASA is also effective in the prophylaxis of thrombotic events^23^ and risk involved with its use has already been widely studied, it would seem appropriate to consider its application as a secondary prophylaxis of thrombotic events in patients with multisystemic viral endotheliitis (in initial stage, mild COVID-19).

**FIGURE 1:**
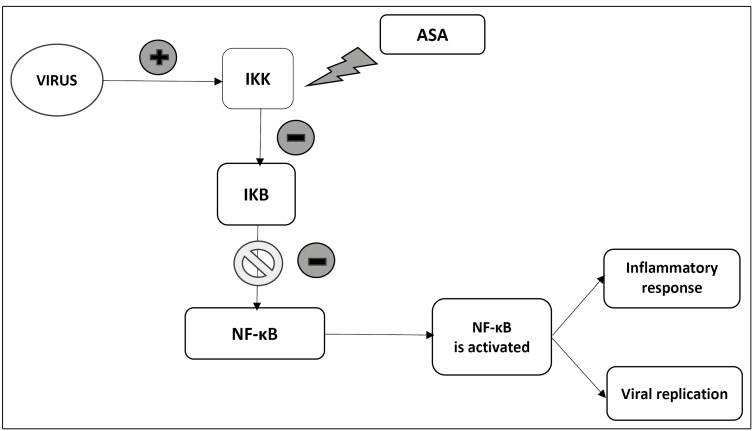
Activation of NF-κB and its effects on the body. ASA and other salicylates block this mechanism by inactivating IKK, which is a key factor in this process. **ASA:** acetylsalicylic acid; **IKK:** IκB kinase; **NF-κB:** *nuclear factor*-*kappa b;* **IκB:** inhibitor of NF-κB.

Another important effect of ASA that may corroborate its possible efficacy for the treatment of COVID-19 is its antiviral action, mediated by the salicylate component^4^. Gurbel et al. reported this possible benefit of ASA for COVID-19, given that this medication inhibits IκB kinase (IKK), which is a factor for viral replication and the inflammatory response (cytokine storm)^4^. Viruses inactivate IκB, the endogenous inhibitor of *nuclear factor*-*kappa b (*NF-κB*)* viruses, by activating IKK, which phosphorylates IκB^4^ (Figure 1). This results in an antiviral effect promoted by ASA and salicylates^4^. Hence, it is possible that ASA is beneficial in the treatment of COVID-19 in two ways by combating thrombosis or the virus itself.

We formulated a hypothesis that statins play a role in preventing and reducing the endotheliitis present in COVID-19 and in impeding the progression of the disease to its most severe form. Several studies have already demonstrated that statins are capable of not only reducing *low*-*density lipoprotein cholesterol* but also of promoting beneficial pleiotropic effects on the endothelium, such as decreasing the degree of inflammation, oxidative stress, and reducing pro-inflammatory mediators, such as interleukin-1, interferon gamma, and interleukin-6^5^ ^,^ ^6^. Statins also increase the production of nitric oxide and number of circulating endothelial progenitor cells and inhibit endothelial cell apoptosis^5^ ^,^ ^6^. The Massachusetts General Hospital treatment protocol for COVID-19, published in May 2020, indicated the use of high-potency statins in the therapeutic approach for all patients, considering the probable role of this class of drugs against the virus^24^ ^,^ ^25^.

Thus, our hypothesis is that statins associated with ASA may have acted together to prevent endothelial injury in our patients and could possibly have the same effect in those cases that tend to progress into more severe forms.

Because this study is only a case series, with no control group and a low number of patients, it is not possible to provide definitive conclusions or recommendations with regard to using ASA with or without statin as a therapy for COVID-19. Moreover, we did not describe standardized doses in a similar time period for the patients involved or that the patients also used other concomitant therapies, which may have influenced the results. 
